# Non-alcoholic fatty liver disease is associated with decreased bone mineral density in upper Egyptian patients

**DOI:** 10.1038/s41598-023-31256-w

**Published:** 2023-03-16

**Authors:** Amro M. Hassan, Mustafa Ahmed Haridy, Mohamed Z. Shoaeir, Tarek M. Abdel-Aziz, Mohamed Khairy Qura, Eglal M. Kenawy, Tarek Mohamed M. Mansour, Sameh Salaheldin Elsayed, Wael Esmat Ali, Mona Mohamed Abdelmeguid, Muhammad Abdel-Gawad

**Affiliations:** 1 Hepatology, Gastroenterology and Infectious Diseases Department, Faculty of Medicine, Al-Azhar University, Assiut, Egypt; 2 Rheumatology and Rehabilitation Department, Faculty of Medicine, Al-Azhar University, Assiut, Egypt; 3 Internal Medicine Department, Faculty of Medicine, Al-Azhar University, Assiut, Egypt; 4 Radiodiagnosis Department, Faculty of Medicine, Al-Azhar University, Assiut, Egypt; 5 Medical Biochemistry Department, Faculty of Medicine, Al-Azhar University, Assiut, Egypt; 6 Clinical Pathology Department, Faculty of Medicine, Al-Azhar University, Assiut, Egypt

**Keywords:** Gastroenterology, Rheumatology

## Abstract

Nonalcoholic fatty liver disease (NAFLD) has been linked with a number of extra hepatic diseases and could be a potential risk factor of decreasing bone mineral density. To determine whether Upper Egyptian patients with NAFLD are at risk of developing osteoporosis. Cross sectional study was done on a total 100 individuals; 50 patients diagnosed with NAFLD (based on ultrasound imaging) crossed-matched with 50 individuals without NAFLD based on age, sex and body mass index. Bone mineral density, serum calcium and phosphorus levels, serum parathyroid hormone, serum vitamin D and fasting insulin level were assessed. Osteoporosis was prevalent in NAFLD patients versus to controls (19/50 vs. 0/50; *P* < 0.001). There was significant decrease in bone mineral density in NAFLD patients than controls (− 2.29 ± 0.4 vs. − 1.53 ± 0.1; *P* < 0.001). There was a statistical significance decrease in serum vitamin D and calcium levels in NAFLD patients than controls. Furthermore, vitamin D levels in the NAFLD group was a predictor for osteoporosis (OR 0.614; 95% CI 0.348–0.825). Patients with NAFLD tend to have a significant decrease in bone density, vitamin D, and serum calcium levels than controls.

## Introduction

Nonalcoholic fatty liver disease (NAFLD) has emerged as noninfectious liver disease affecting between 17 and 33% in the general population and 75% in obese and/or diabetic individuals worldwide^1^. NAFLD is not confined to adult but it occurs in children and adolescents. Prevalence report from Egypt estimates NAFLD among school children was 15.8% in cross-sectional study^2^.

The pathological spectrum of NAFLD ranges from simple steatosis to nonalcoholic steatohepatitis (NASH) that ultimately may progress to fibrosis and cirrhosis which may be complicated by hepatocellular carcinoma^3,4^.

NAFLD has been linked with extra hepatic diseases as cardiomyopathy, cardiac arrhythmias, chronic kidney disease, type 2 diabetes mellitus (T2DM) and obstructive sleep apnea^3,5,6^. Moreover, NAFLD has been associated with increased risk of extra hepatic cancer including gastrointestinal, urinary, lung, breast, and gynecological cancers^7^.

Osteoporosis (OP) is a systemic skeletal disease characterized by low bone mineral density (BMD) and may be associated with pathological fracture^8^. In developed countries, OP affecting 9–38% of women and 1–8% of men aged > 50 years^9^.

Diagnosis of OP is based on dual-energy X-ray absorptiometry (DEXA)^10^. There are multiple sites that could be used to assess bone mineral density including hip, spine and wrist. In spite of these multiple sites, DEXA scanning of the hip or spine is validated by world health organization (WHO)^11^.

About 12–55% of patients with liver cirrhosis have imminent risk of vertebral fractures so patients with chronic liver disease need to be screened by DEXA scan for early detection of OP as vertebral fractures are usually asymptomatic^12,13^.

Several cross sectional studies have evaluated association between NAFLD and lower BMD. Unfortunately, the results of these studies were conflicting as some studies have identified a significant association between NAFLD and low BMD^14,15^ and other studies showed no significant associations between NAFLD and low BMD^17,18^.

Some Studies showed that NAFLD may contribute in the pathophysiology of bone demineralization and OP via production of multiple pro-inflammatory cytokines, tumor necrosis factor α (TNF-α), pro-oxidant mediators, bone-influencing molecules and/or via the direct effect on hepatic and insulin resistance^12,19^.

As association between NAFLD and low BMD is still a matter of controversy, so the study aimed to evaluate bone mineral density, serum calcium and vitamin D in patients with NAFLD to determine if patients who have NAFLD are at risk of developing OP.

## Patients and methods

This cross section study was done in Hepato-Gastroentrology and rheumatology departments, Al-Azhar Assiut University hospital, from February 2019 to December 2020 to determine if patients who have NAFLD are at risk of OP.

A total 100 individuals were enrolled in the study; 50 patients diagnosed by ultrasound to have NAFLD and 50 crossed matched individuals without NAFLD based on age, sex and BMI.

The study was approved by ethical committee of Al-Azhar Assiut faculty of medicine and an informed written consent was signed by every individual before being enrolled in the study. The study was conducted in accordance with ethical principles of the World Medical Association Declaration of Helsinki.

### Inclusion criteria

Any individual diagnosed by ultrasound to have (NAFLD) and aged ≥ 18 years.

### Exclusion criteria

Individual with any one of the following criteria were excluded from the study: (1) aged ≤ 18 years, (2) any liver disease that could lead to NAFLD such as viral hepatitis, autoimmune liver diseases, alcohol consumption, Wilson’s disease, hemochromatosis, (3) recent exposure to hepatotoxic drugs within 6 months or drugs containing or affecting vitamin D level, (4) diabetes mellitus, (5) renal disease (6) Pregnant or lactating women.

### Investigatory work-up

Eligible individuals (cases and controls) were admitted to Hepato-Gastroentrology and rheumatology departments, et al.-Azhar Assiut University Hospital and full history taking, clinical examination and BMI were assessed for every individual.

After midnight fasting, eligible individuals were assessed by the following laboratory tests and imaging studies: complete blood count (CBC), liver function tests (serum bilirubin, AST, ALT, albumin and INR), renal function test (urea, creatinine and serum uric acid), fasting blood sugar (FBS), cholesterol, triglycerides and erythrocyte sedimentation rate (ESR).

Serum calcium and phosphorus levels were assessed by colorimetric method using (spinreact, S.A.U., Spain) and (5010 chemistry photometer, Germany) and reference range for serum calcium is 8.0–10.5 mg/dl and 2.5–5 mg/dl for phosphorus.

Serum parathyroid hormone level was assessed by ELISA using (Human PTH ELISA Kit, Bioassay technology laboratory) produced by (Shanghai Korian Biotech Co., Ltd, China) and (Robonik ELISA plate reader, India).

Serum 25-OH Vitamin D3/D2 level was assessed by ELISA using (ORGENTEC Diagnostika GmbH, Germany) and (Robonik ELISA plate reader, India).

Fasting insulin level was assessed by ELISA using (human insulin enzyme immunoassay test Kit , prechek Bio Inc , USA) and (Robonik ELISA plate reader , India) and Homeostasis Model Assessment of Insulin Resistance (HOMA-IR) was calculated by using the following equation: HOMA-IR = fasting insulin (micro unite/ml) × fasting blood glucose (mmol/ml)/22.5^20,21^.

Pelvi-abdominal ultrasound examination for eligible individuals was done after a midnight fasting using a B-mode convex probe US equipment (Esaote ID, CE0051; Technos, Genoa, Italy) with a 4.5–7 MHz to assess severity of fatty liver and NAFLD was graded according to echogenicty of the liver^22^ into Grade I: minimal diffuse increase in hepatic echogenicty in which the liver appears bright compared with the cortex of the kidney with normal visualization of diaphragm and borders of intrahepatic vessel, Grade II: moderate diffuse increase in hepatic echogenicty with slightly impaired visualization of diaphragm and intrahepatic vessels, Grade III: marked increase in hepatic echogenicty which obscures visualization of intrahepatic vessels and diaphragm.

Bone mineral density (BMD) at lumbar spine was measured by dual energy X-ray absorptiometry (DEXA) (DEXA scan lunar DPX-NT 2013 made in USA by General Electric) and according to the World Health Organization (WHO) criteria for BMD, OP in adult is defined by a T score less than -2.5 and osteopenia is defined by a T score between − 1 and − 2.5^11,23–25^***.***

### Statistical analysis

The statistical analysis was performed using Windows 10 SPSS version 22 (IBM SPSS Inc., Chicago, Illinois, USA)) program. Normally distributed data were presented as mean ± standard deviation (SD) and categorical data were expressed as number and percentage. The Student’s *t* test was performed for continuous variables and categorical variables were compared by the chi-square (χ2) and Fisher's exact tests. Regression analysis was done to predict the independent associated factors that may affect BMD in patients with NAFLD. *P* value of < 0.05 was considered as statistical significance.

## Results

A total of 100 individuals were included in the study. The baseline and laboratory characteristics of individuals with NAFLD (cases) and individuals without NAFLD (controls) is shown in (Table 1).

**Table 1:** Baseline and Laboratory characteristics of studied groups.

Parameters	Cases (N = 50) Mean ± SD	Controls (N = 50) Mean ± SD	*p* value
Age (years) Mean + standard deviation (SD)	45.40 ± 12.5	45.12 ± 11.5	0.907
Male	28 (56%)	30 (60%)	0.420
Female	22 (44%)	20 (40%)	
Nonsmoker	37 (74%)	38 (76%)	0.817
Smoker	13 (26%)	12 (24%)	
Body mass index(BMI)	27.85 ± 5.4	27.77 ± 5.5	0.941
Hemoglobin concentration (g/dl)	12.79 ± 1.2	12.71 ± 1.3	0.41
White blood cells *103/µL	38.08 ± 10.6	36.09 ± 11.4	0.37
Red blood cells (106/mm3)	4.91 ± 0.9	5.01 ± 1.0	0.9
Platelet *103/µL	270.91 ± 13.3	288.46 ± 16.2	0.76
Mean corpuscular volume (M CV) (fl)	83.95 ± 9.0	83.78 ± 8.9	0.072
Mean Corpuscular Haemoglobin (MCH) (pg)	28.43 ± 4.1	27.65 ± 1.8	0.13
Triglycerides (mg/dl)	162.12 ± 12.3	163.66 ± 10.4	0.929
Total cholesterol (mg/dl)	183.22 ± 8.4	176.64 ± 7.5	0.560
Erythrocyte sedimentation rate (ESR) (mm/hr)	21.44 ± 1.8	22.60 ± 1.9	0.661
Alanine transferase (IU/L)	31.52 ± 2.5	23.62 ± 1.4	0.003
Aspartate transferase (AST) (IU/L)	30.58 ± 2.1	28.60 ± 2.3	0.252
Albumin (g/L)	4.17 ± 0.5	4.15 ± 0.5	0.868
Total Protein (g/dl)	7.17 ± 0.7	7.29 ± 0.7	0.382
Total Bilirubin (mg/dl)	0.64 ± 0.1	0.65 ± 0.1	0.929
Direct Bilirubin (mg/dl)	0.22 ± 0.1	0.21 ± 0.1	0.820
International normalized ratio (INR)	1.10 ± 0.1	1.11 ± 0.2	0.770
Urea (mg/dl)	34.22 ± 10.8	31.34 ± 10.5	0.179
Creatinine (mg/dl)	0.90 ± 0.1	0.82 ± 0.1	0.142
Fasting blood sugar (FBG) (mg/dl)	98.94 ± 16.3	97.14 ± 15.6	0.589
Fasting Insulin (mIU/L)	4.50 ± 0.4	4.03 ± 0.3	0.380
HOMA-IR, median (range)	0.96 (0.329–2.24)	0.827 (0.19–3.26)	0.326

*HOMA-IR* homeostasis model assessment of insulin resistance.

Our study showed no significant difference between cases and controls regarding to age, sex, smoking, BMI and the baseline and laboratory characteristic of studied groups. Moreover, cases had elevated ALT more than controls with significant P-value 0.003 (Table 1).

Among 50 patients enrolled in the study, 41 (82%) patients had grade I fatty liver and 9 (18%) patients had grade **≥** II (Table 2).

**Table 2:** Ultrasound characteristics of studied groups.

Parameters	Cases (N = 50) Mean ± SD	Controls (N = 50) Mean ± SD	*p* value
US /Fatty liver grade 0	0 (0%)	50 (100%)	< 0.001
US /Fatty liver grade I	41 (82%)	0 (0%)	
US /Fatty liver grade ≥ II	9 (18%)	0 (0%)	

*US* ultrasound.

As regarded to factors affecting bone density, the study showed a significant difference in PTH, vitamin D, and serum calcium between cases and controls group (Table 3).

**Table 3:** Factors affecting bone density among studied groups.

Factors affecting bone density	Cases (N = 50) Mean ± SD	Controls (N = 50) Mean ± SD	*p* value
PTH (pg/ml)	56.27 ± 7.8	32.48 ± 9.1	0.002
Vitamin D (ng/ml)	28.94 ± 7.5	61.50 ± 14.1	< 0.001
Serum Ca (mg/dl)	8.38 ± 0.6	9.86 ± 0.7	< 0.001
Serum P (mg/dl)	4.74 ± 0.9	4.87 ± 0.8	= 0.414

*PTH* parathyroid hormone, *Ca* calcium, *P* phosphorus.

DEXA scan results showed a significant difference between cases (− 2.29 ± 0.4) and controls (− 1.53 ± 0.1) with *p* value < 0.001 (Table 4).

**Table 4:** Dual energy X ray absorptiometry among studied groups.

	Cases (N = 50) Mean ± SD	Controls (N = 50) Mean ± SD	*p* value
DEXA Scan (BMD) T-score	− 2.29 ± 0.4	− 1.53 ± 0.1	< 0.001

*DEXA* dual energy x-ray absorptiomet ry.

Our results showed a significant difference in bone density between cases and controls. Moreover, OP was more prevalent in cases than controls group with *p* value < 0.001. Among the NAFLD group, 19 patients had OP (16 of them had grade I fatty liver and 3 of them had grade ≥ II fatty liver), 28 patients had osteopenia (22 of them had grade I fatty liver and 6 had grade **≥ **II fatty liver) 3 patients had normal bone density (Table 5).

**Table 5:** Relationship between degree of fatty liver and degree of bone density.

Variable	Osteoporosis (n = 19)	Osteopenia (n = 68)	Normal (n = 13)	*p* value
Control	0 (0%)	40 (58.8%)	10 (76.9%)	< 0.001
NAFLD	19 (100%)	28 (41.2%)	3 (23.1%)	
Grade of fatty liver				
0	0 (0%)	40 (58.8%)	10 (76.9%)	< 0.001
I	16 (84.2%)	22 (32.4%)	3 (23.1%)	
> I	3 (15.8%)	6 (8.8%)	0 (0%)	

In our study, we performed logistic regression model analysis to determine predictors of OP among the NAFLD group. We found among patients with NAFLD, serum vitamin D level was statistically significant predicts OP (OR 0.614; 95% CI 0.348–0.825) (*P* < 0.001). Also female gender, BMI, bilirubin, serum calcium, serum phosphorus PTH, fasting insulin, and insulin resistance were statistically significant predictors of OP among the NAFLD group (Table 6).

**Table 6:** Predictors of Osteoporosis among the NAFLD Sample: Logistic Regression Model.

Variable (per unit increase)	Univariate		Multivariate	
	OR (95% CI)	*P* value	OR (95% CI)	*P* value
Age/years	1.023 (0.976–1.073)	0.344	1.013 (0.981–1.062)	= 0.547
Sex (Female)	3.600 (1.086–7.932)	0.036	2.288 (1.147–6.655)	= 0.018
Smoker	3.516 (1.102–9.446)	0.021	3.057 (1.066–5.126)	= 0.031
BMI	1.185 (1.006–2.008)	0.048	2.258 (1.287–4.916)	= 0.040
Bilirubin (mg/dl)	2.521 (1.033–8.361)	0.044		
Serum Ca (mg/dl)	0.549 (0.212–0.769)	0.019	0.180 (0.043–0.756)	= 0.019
Serum P (mg/dl)	0.893 (0.529–0.994)	0.031	0.741 (0.425–0.921)	= 0.044
PTH (pg/ml)	1.098 (1.008–1.120)	0.041	1.020 (1.002–1.039)	= 0.029
Fasting Insulin	1.313 (1.060–1.627)	0.013	1.868 (1.218–2.866)	= 0.004
HOMA-IR	3.056 (1.244–7.507)	0.015		
Vit. D Level (ng/ml)	0.614 (0.348–0.825)	< 0.001	0.268 (0.018–0.446)	= 0.011

*OR* odd ratio, *CI* confidence interval.

## Discussion

Large numbers of cross sectional studies have identified a significant association between NAFLD and low BMD, but this association remains a matter of controversy^14,18^. One meta-analysis showed no significant difference in BMD between patients with NAFLD and controls^26^. Another meta-analysis showed NAFLD was associated with osteoporotic fracture, but not associated with low BMD^27^***.*** However, recent meta-analysis done by Mantovani et al. found that NAFLD was significantly associated with low BMD in children and adolescents^28^.

Our study showed a significant difference in bone density between patients with NAFLD and controls. Our results agree with Xia et al. who found that patients with NAFLD were significantly associated with low BMD^29^. Lee et al. reported a positive association between NAFLD and lumbar spine BMD in postmenopausal females^17^. Moreover, in Shen et al. cohort study showed that males and females patients with NAFLD were associated with increased risk of low BMD^30^.

Our results showed that osteoporosis was more prevalent in patients with NAFLD compared to controls with *p* value < 0.001. Moreover among NAFLD group, 19 patients had osteoporosis and 28 patients had osteopenia. Our result is supported by meta-analysis study done by Pan et al.^31^ who reported that prevalence and risk of OP or osteoporotic fracture was significantly associated with NAFLD group than in controls. Also, Loosen et al.^32^ reported that incidence of OP was significantly higher in the NAFLD patients (6.4%) compared to controls (5.1%) with *p* value < 0.001). Moreover, our study agrees with Pirgon et al.^33^ who indicated that NAFLD has a noxious effect on BMD in adolescents and was correlated with increased insulin resistance. Another cohort study performed by Chen et al.^34^ showed increase risk of OP 1.35 times in patients with NAFLD than individuals without NAFLD. Also Li et al.^16^ showed that NAFLD was significantly associated with history of osteoporotic fractures in middle-aged and elderly Chinese men.

Our results can be interpreted in light that factors affecting bone mineral density including serum vitamin D and serum calcium were significantly lower in our studied patients with NAFLD than controls. Our results agree with Targher et al. who reported a potential link between decreased serum vitamin D and low BMD in patients with NAFLD^19^. Moreover, many studies proved that patients with NAFLD have lower levels of serum vitamin D than controls which lead to decrease BMD and increase risk of fractures^35–38^. Our study showed that patients with NAFLD had increased level of PTH compared to controls which indicates that patients with NAFLD had low BMD and OP.

The association between hypovitaminosis D and NAFLD have been found in many diseases such as metabolic syndrome and obesity^39,40^. But in observational studies like our study, it is difficult to judge if NAFLD is a cause or result to hypovitaminosis D. Moreover, association between hypovitaminosis D and NAFLD could be accidental without actual relations between them. Consequently, the relationship between hypovitaminosis D and NAFLD needs prospective randomized controlled trials to compare development of NAFLD in patients with hypovitaminosis D versus healthy subjects and to assess effect of vitamin D supplementation on regression of NAFLD.

In our study, we have performed logistic regression model analysis to determine predictors of osteoporosis among patients with NAFLD. We found that serum vitamin D levels was a statistically significant predictor for OP among NAFLD patients (OR 0.614; 95% CI 0.348–0.825) (*P* < 0.001).

Insulin resistance is considerable risk factor for NAFLD^4,41^ and insulin resistance and high fasting serum insulin may be associated with increased risk of low BMD^42^***.***

Although most people with NAFLD have metabolic dysfunction such as diabetes mellitus, but we excluded diabetic patients as diabetes mellitus deteriorates bone strength and increase susceptibility to bone fracture and this could affect results of our study^43,44^.

In our study, 10 patients had HOMA-IR more than 2, nine of them had decreased BMD (osteoporosis and osteopenia) and only one patient had significant insulin resistant (HOMA-IR more than 2.7). The patient who had HOMA-IR more than 2.7 also had osteopenia.

In our study logistic regression model analysis showed that fasting insulin and insulin resistance were statistical significant predictors of OP among the NAFLD group. Our result agrees Filip et al.^8^ who found that patients with NAFLD are associated with insulin resistance which is risk factors for low BMD.

## Conclusion

Our study showed significant decreased in bone density and osteoporosis in patients with NAFLD compared to controls. Also serum vitamin D and serum calcium were significantly deceased in patients with NAFLD while level of PTH was increased in patients with NAFLD than controls which indicate that patients with NAFLD have potential risk of developing low BMD and OP. Additional further prospective studies are needed to determine the relationship between NAFLD and risk of low BMD and OP.

### Study limitations and future recommendations

This study had some limitations. The first limitation was the small sample size so we recommend further studies with large sample size with different risk factors to determine the relationship between NAFLD and the risk of low BMD and osteoporosis. Second, ultrasound had been used to determine whether individuals had NAFLD or not and its degrees, instead of liver biopsy which is the gold standard in diagnosis of fatty liver. Third, minimal steatosis (NAFLD grade 1) might be missed by using ultrasound. Fourth, further studies are recommended to screen BMD in patients with NAFLD who have diabetes mellitus as most patients with NAFLD have metabolic dysfunction as diabetes mellitus or insulin resistance. Moreover, in this study we screened only lumbar spine to detect BMD in patients with NAFLD and individuals without NAFLD and we recommend further studies for screening BMD of other vulnerable sites of fracture such as neck of the femur.

## Data Availability

Regarding to the datasets used and/or analyzed during the current study, the datasets used and/or analyzed will be available from the corresponding author on reasonable request.
